# Genome-scale community modelling elucidates the metabolic interaction in Indian type-2 diabetic gut microbiota

**DOI:** 10.1038/s41598-024-63718-0

**Published:** 2024-07-27

**Authors:** Satyajit Beura, Pritam Kundu, Amit Kumar Das, Amit Ghosh

**Affiliations:** 1 Department of Bioscience and Biotechnology, Indian Institute of Technology Kharagpur, Kharagpur, West Bengal 721302 India; 2 School of Energy Science and Engineering, Indian Institute of Technology Kharagpur, Kharagpur, West Bengal 721302 India; 3 P.K. Sinha Centre for Bioenergy and Renewables, Indian Institute of Technology Kharagpur, Kharagpur, West Bengal 721302 India

**Keywords:** Biochemical networks, Computer modelling

## Abstract

Type-2 diabetes (T2D) is a rapidly growing multifactorial metabolic disorder that induces the onset of various diseases in the human body. The compositional and metabolic shift of the gut microbiota is a crucial factor behind T2D. Hence, gaining insight into the metabolic profile of the gut microbiota is essential for revealing their role in regulating the metabolism of T2D patients. Here, we have focused on the genome-scale community metabolic model reconstruction of crucial T2D-associated gut microbes. The model-based analysis of biochemical flux in T2D and healthy gut conditions showed distinct biochemical signatures and diverse metabolic interactions in the microbial community. The metabolic interactions encompass cross-feeding of short-chain fatty acids, amino acids, and vitamins among individual microbes within the community. In T2D conditions, a reduction in the metabolic flux of acetate, butyrate, vitamin B5, and bicarbonate was observed in the microbial community model, which can impact carbohydrate metabolism. The decline in butyrate levels is correlated with both insulin resistance and diminished glucose metabolism in T2D patients. Compared to the healthy gut, an overall reduction in glucose consumption and SCFA production flux was estimated in the T2D gut environment. Moreover, the decreased consumption profiles of branch chain amino acids (BCAAs) and aromatic amino acids (AAAs) in the T2D gut microbiota can be a distinct biomarker for T2D. Hence, the flux-level analysis of the microbial community model can provide insights into the metabolic reprogramming in diabetic gut microbiomes, which may be helpful in personalized therapeutics and diet design against T2D.

## Introduction

The human gut microbiota is the collection of a large number of microbes present in the human digestive tract. It primarily consists of bacteria, archaea, fungi, viruses, and other microbes^1^. The gut microbiota performs a multitude of essential functions within the human body, including nutrient metabolism, and contributes to maintaining the host’s physiological homeostasis through its metabolic activities^2^. The gut microbiota provides essential health benefits to the host by bolstering metabolism, including short-chain fatty acid (SCFAs) and vitamin metabolism, bile acid transformation, amino acid (AA) synthesis, and fermentation of complex biomolecules^3,4^. The human gut microbial population can have unique composition according to food habits, lifestyle, and demographic location^5,6^. For the Indian population, the gut is dominated by firmicutes and bacteroidetes microbial phyla, with a coverage of 62 and 24%, respectively^7^. Within these phyla, *Prevotella* sp., *Ruminococcus* sp., *Bifidobacterium* sp*., and Lactobacillus* sp. are found to be the most dominant microbial taxa, which are associated with carbohydrate and fiber-rich Indian diet^8–12^. Additionally, *Faecalibacterium sp.* and *Methanobrevibacter sp.* are vital gut microbes that help in the fermentation of dietary fibers and contribute towards energy metabolism and immune response regulation^13^. *Faecalibacterium sp.* produces different SCFAs like butyrate, acetate, and formate, whereas *Methanobrevibacter sp.* can produce methane as a fermentation product in the human gut microbiota. SCFAs serve as energy substrates for the host^14^, providing an additional 10% of daily dietary energy that can be utilized for various metabolic processes^15^. Approximately 70% of ATP production in the colon is attributed to SCFAs synthesized by microorganisms, with butyrate being the major energy source for colonocytes^16^. Therefore, disruption in the gut microbial population and their metabolic profile can hinder human metabolism, potentially leading to various health complications like colorectal cancer (CRC), obesity, T2D, and inflammatory bowel disease (IBD)^17^.

The association of T2D with the varied composition of the human gut microbiome has been highlighted in different metagenomics studies. One prominent finding is the decrease in microbial diversity in the human gut microbiota of Indian T2D individuals^18^. In the case of T2D, it has been seen that the abundance of *Prevotella* sp., *Lactobacillus* sp., *Ruminococcaceae* sp.*, Lachnospiraceae* sp.*, Faecalibacterium* sp., and *Methanobrevibacter* sp. is heavily altered in comparison to healthy individuals, which can be a distinctive biomarker for T2D in the Indian population^18–21^. Specifically, studies have found a reduction in butyrate-producing bacteria, like *Roseburia* sp. and *Faecalibacterium prausnitzii*, which are anti-inflammatory in nature and maintain gut health^18^. Conversely, an increase in pro-inflammatory bacterial species, such as *Escherichia coli* and *Enterobacter* species, has been observed in individuals with T2D^22^. Additionally, the human gut microbes linked to a plant-based diet, namely *Prevotella copri* and *Lactobacillus ruminis*, exhibit an altered abundance in Indian T2D patients^19,23^. Furthermore, metabolomics studies have highlighted the significance of metabolites in the onset of T2D, particularly the SCFAs such as butyrate, propionate, and acetate^24,25^. SCFAs are essential for maintaining gut barrier function, regulating energy metabolism, and influencing insulin sensitivity. The imbalance of SCFA production in individuals with T2D suggests a disruption in gut microbial metabolic activity. Although these metagenomics and metabolomics studies have made significant advancements in identifying altered gut microbial populations and varied metabolic profiles in T2D, a comprehensive understanding of the metabolic intercommunication within the gut microbiota and their impact on T2D patients needs to be explored.

Understanding the complex gut microbiota is challenging; however, explicit mathematical modelling offers the promise of in silico evaluation and analysis of biological phenomena. As computational capacity continues to advance and high-throughput multi-omics data becomes more available, in silico genome-scale models (GEMs) were reconstructed to explore the metabolic activity of individual microbes^26–28^. A genome-scale model (GEM) is a mathematical representation of the metabolic reactions occurring within a cell using the information related to gene-protein-reaction (GPR) association. Further, the species-specific biochemical data on cellular metabolism, accessible through various databases and literature, can also be leveraged to enhance the accuracy of model predictions. Furthermore, to understand the multi-species system, an extension of GEM, known as the genome-scale community metabolic model, offers the opportunity to explore microbe-microbe and microbe-host interaction^29,30^. Multiple tools for community metabolic modelling have been developed to effectively capture the interactions within multi-species microbial communities, including MICOM, OptCom, MIGRENE, MM Toolbox, SteadyCom, etc.^30–34^. The community modelling approach has been widely implemented to explore the altered behavior of the human gut microbiota under multiple health and disease conditions^31,34–36^. A genome-scale community metabolic model was developed for 616 human gut microbiome samples associated with CRC patients using the MM Toolbox^36^. This modelling approach uncovers the significant effects of *Fusobacterium* sp. on butyrate production, highlighting the crucial connection between gut microbiota and CRC. Another study employed the community metabolic modelling approach to investigate the altered gut microbiome metabolism among 25 malnourished children in Bangladesh, using COBRA Toolbox^37^. The study illustrates that a reduction in microbial diversity leads to a decrease in the synthesis of crucial AAs such as L-lysine, L-histidine, L-tryptophan, L-isoleucine, and L-valine within the gut microbiota, which is associated with infant malnutrition. Similarly, a community metabolic model of 108 human gut microbiome samples was constructed to reveal the altered metabolic activity of IBD patients^38^. The finding showed elevated hydrogen sulfide (H2S) production, indicating a correlation with the abundance of Gammaproteobacteria genera, resulting in inflammation within the gastrointestinal environment. Recently, the impact of metformin therapy on T2D gut microbiome metabolism was studied using the MIGRENE toolbox^34^. Following the treatment, an altered abundance of *Akkermansia muciniphila*, *Alistipes obesi*, *E. coli*, and *F. prausnitzii* was observed in the gut microbiota, leading to variations in the production of H2S, SCFAs, and branched-chain amino acids (BCAAs). These changes in the metabolic profile are associated with insulin sensitivity and sugar metabolism. Hence, the community metabolic modelling can explore the human gut microbiota’s altered metabolic behavior under Indian T2D conditions compared to healthy controls. Furthermore, community modelling also aids in personalized diet and therapeutics, offering valuable insights and strategies for tailoring interventions based on the disease.

Our study aims to comprehend the interlink between the metabolic role of the gut microbiota and Indian T2D. We reconstructed the in silico metabolic models of the selected human gut microbes associated with T2D. These microbial models were integrated to construct the community metabolic model. The abundance of individual microbes in healthy and T2D gut environments was considered to constrain the community model, which can reflect the respective gut microbiota. Further, the biomass production of the community metabolic model was optimized in a gut microbiota environment by proving the average human diet. The microbial community in T2D and healthy gut environment were compared to explore the altered metabolic profile and inter-microbial metabolic dependency between the individual microbes. We show that several metabolic components like SCFAs, AAs, vitamins, CO_2_, and bicarbonate were exchanged between the individual microbes present in the community. The study also helps decipher the altered flux flow in biochemical pathways, including glucose, amino acid, SCFA, and vitamin metabolism, within the individual entities of the microbial community in different gut environments and identify crucial connections between the gut microbiome and T2D.

## Results

### Reconstruction of a genome-scale metabolic model

**Figure 1:**
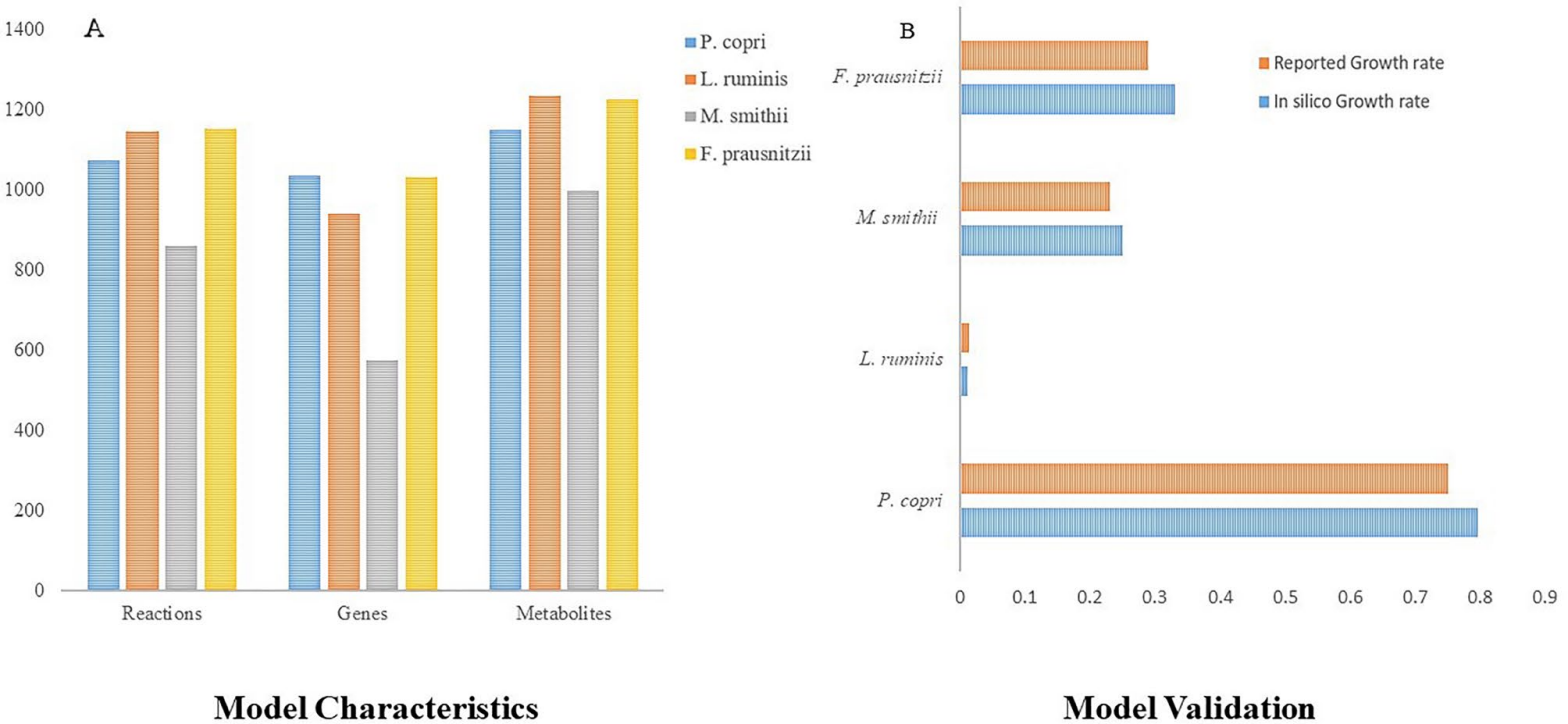
Model characteristics and validation. (**a**) The plot represents the total number of genes, metabolites and reactions present in all individual models. (**b**) Model validation plot shows the comparison of the experimental growth rate and in silico growth rate of all individual models. The horizontal axis represents the growth rate (h^−1^), and the vertical axis shows the individual microbes.

In the Indian T2D gut microbiota, species like *L. ruminis*, *P. copri*, *M. smithii,* and *F. prausnitzii* showed an altered abundance and exhibited changes in the metabolic activities associated with carbohydrate utilization and SCFA production^18^. To explore microbial metabolic behavior associated with Indian T2D, genome-scale metabolic modelling of *L. ruminis*, *P. copri, M. smithii*, and *F. prausnitzii* was implemented. Initially, the draft models were reconstructed for each microbial species based on the respective genome annotation profile. The draft GEMs of *P. copri*, *L. ruminis*, *M. smithii*, and *F. prausnitzii* comprised 15, 4, 9, and 18 unbound reactions, respectively. These reactions involve thermodynamically infeasible cycles (TIC) and do not result in the consumption or production of any metabolites^29^. In order to identify these TICs in our GEMs, we disabled all nutrient uptake reactions for the microbes and performed flux variability analysis (FVA). FVA determines the flux ranges for each reaction, and those hitting either the lower or upper bound are defined as unbound reactions^39^. In order to eliminate the unbound reactions, duplicate reactions were removed, lumped reactions were turned off, and reactions were selectively turned on or off based on available cofactor specificity information from MetaCyc^40^ and ModelSEED reaction databases^41^. In addition to unbounded reactions, the draft GEMs also included unbalanced reactions. Maintaining charge and mass balance in reactions is crucial for enhancing the predictive accuracy of GEMs^42^. In order to maintain elemental and charge balance in biochemical reactions, each metabolite was initially assigned accurate chemical formula and charge by consulting CHEBI (Chemical Entities of Biological Interest)^43^, PubChem, and BiGG Database^44^. Furthermore, the databases (CHEBI, BiGG) were employed to rectify the proton-imbalanced reactions by adding or removing one or more protons on either the reactant or product side. However, the reaction stoichiometry was adjusted for the remaining imbalanced reactions to guarantee that the number of atoms on both sides of the reaction was equal. These corrections led to 56, 49, 16, and 42 balanced reactions in *P. copri*, *L. ruminis*, *M. smithii,* and *F. prausnitzii,* respectively. The model also included orphan and dead-end metabolites lacking proper metabolic pathways, accounting for an average of 2–5% of the total metabolites across all four GEMs. These metabolites were either eliminated from the models or integrated into the relevant metabolic pathway to improve the quality of the model. Moreover, correcting the reversibility of all essential biochemical reactions is necessary to enhance the accuracy of the metabolic network. The correct direction was assigned by adjusting the upper and lower limits for each individual reaction by referring to the ModelSEED database and MetaCyc. Assigning appropriate direction to biochemical reactions greatly enhances the metabolic flux flow through the biochemical pathways. The model exhibited a significant proportion, ranging from 20 to 50%, of blocked reactions across all microbial models. These blocked reactions resulted from the missing reactions in the central metabolic network. The GEMs of *P. copri*, *L. ruminis*, *M. smithii*, and *F. prausnitzii* exhibit reaction gaps, comprising 74, 31, 136, and 89 reactions, respectively. The gaps in the respective metabolic networks were addressed by incorporating the missing biochemical reactions guided by the KEGG pathway database. Curation of the metabolic networks enhances the accuracy, predictive capability, and biological relevance of these models (Fig. 1a). The biomass production of all the curated GEMs was optimized through flux balance analysis (FBA), a mathematical approach for analyzing the flux of biochemical reactions through a metabolic network. Biomass production was considered as the objective function while optimizing the individual microbial models of *P. copri, L. ruminis, M. smithii,* and *F. prausnitzii* using FBA. The biomass equation of the GEMs represents the combination of precursor molecules, which ideally reflect the molecular makeup of the microorganism. The minimal media was used during the optimization of the objective functions of *P. copri* (DSM 18205), *L. ruminis* (ATCC 25644), *M. smithii*, and *F. prausnitzii* which showed an in silico growth rate of 0.79 mmol/gDW/h, 0.012 mmol/gDW/h 0.25 mmol/gDW/h and 0.33 mmol/gDW/h respectively (Fig. 1b). The predicted in silico growth of the individual models showed good agreement with previously reported experimental observations^37,45^ as well as with the AGORA models^46^ (Table S1).

**Figure 2:**
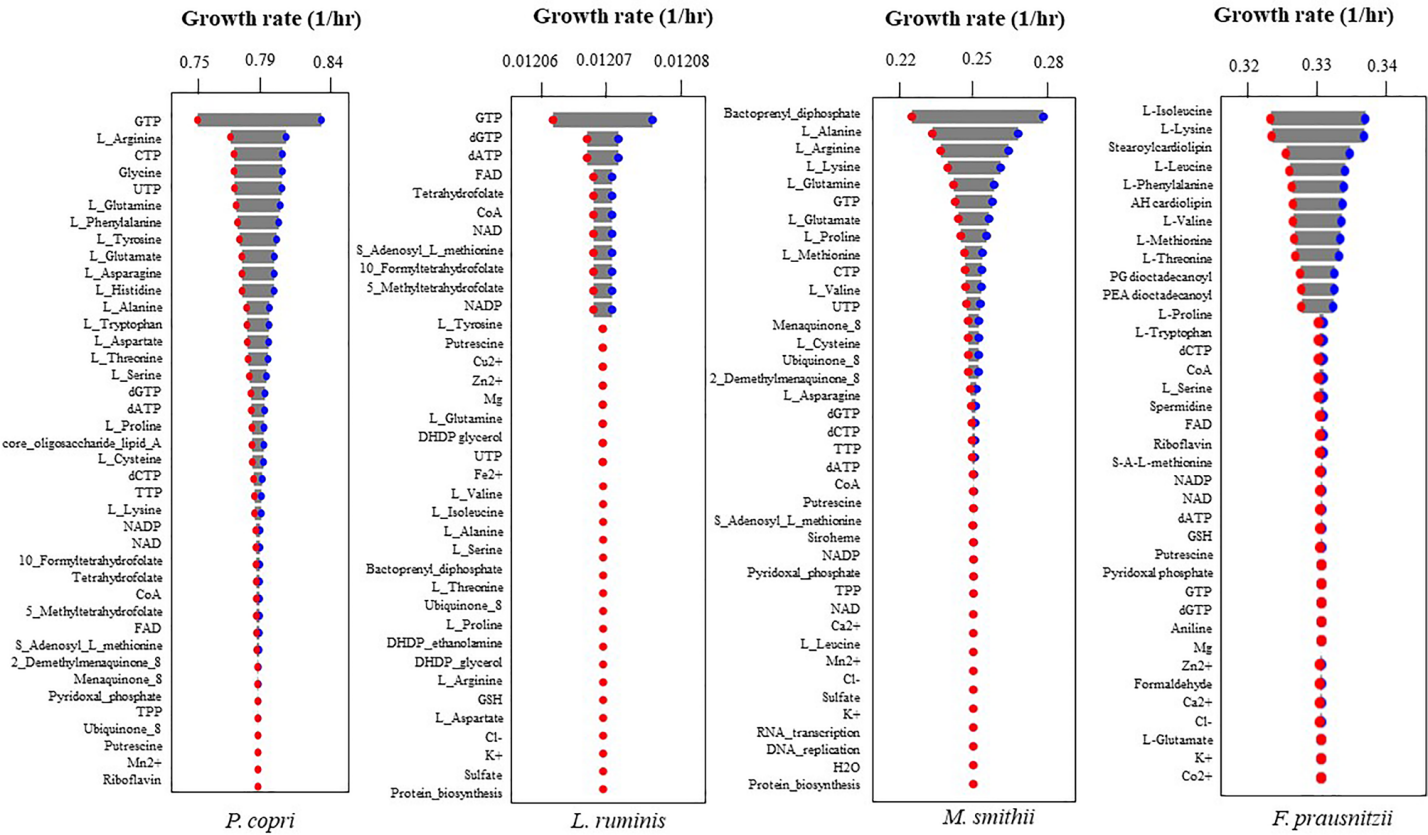
Biomass sensitivity analysis. The plot shows the altered growth rate of the individual models with respect to varied coefficients of the biomass precursor molecules by 20%. The lowest growth rate achieved by altering the coefficient of individual biomass precursor is represented by the red dot, and the highest is in the blue dot. The horizontal axis represents the biomass precursor molecules, and the vertical axis shows the altered microbial growth rate: (**a**) *P. copri*. (**b**) *L. ruminis*. (**c**) *M. smithii*. (**d**) *F. prausnitzii*.

Moreover, a biomass sensitivity assay was performed to check the integrity of the biomass formulation of individual GEMs. During the biomass sensitivity analysis, the coefficient of each biomass precursor was altered by ± 20%, and FBA was performed to estimate the flux through the biomass reaction. The alteration of the biomass precursor by ± 20% showed a change in the biomass formation of 1.92%, 0.65%, 2.57%, and 0.175% in *P. copri*, *L. ruminis, M. smithii*, and *F. prausnitzii,* respectively. The biomass flux was most sensitive towards different AAs, such as arginine, glutamine, alanine, lysine, and methionine, alongside nucleotides like GTP, CTP, and UTP. (Fig. 2). As the amino acids and nucleotides hold a significant fraction of ~ 30% of the biomass equation, the models showed a higher sensitivity towards these precursor molecules (Table S2). Despite the ± 20% alteration in the coefficients of biomass precursor molecules, the growth rate experiences minimal fluctuations between ~ 1 and 3%. This minute alteration in the growth rate stems from flux readjustments within the metabolic network, showing the robust metabolic activity of the individual GEMs.

### Reconstruction of genome-scale community metabolic model

**Figure 3:**
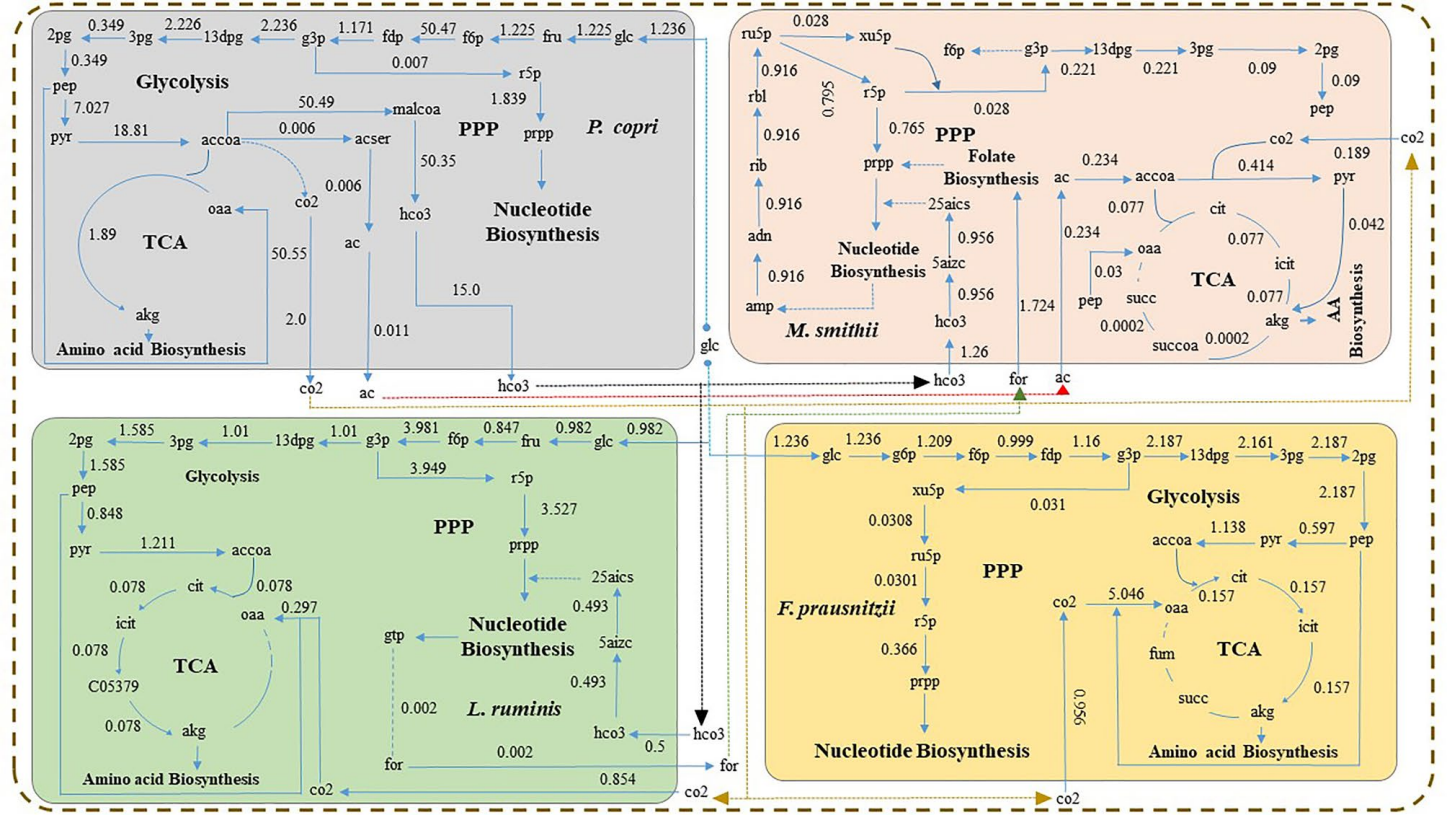
Community-level metabolic interaction between individual gut microbiota in minimal media. This network represents the metabolic interaction between the microbes grown in the minimal media. *P. copri*, *L. ruminis*, *M. smithii*, and *F. prausnitzii* are represented in four different compartments, i.e., blue, green, and red, respectively. Acetate (red), carbon dioxide (yellow), bicarbonate (black), formate (green), and glucose (blue) are exchanged between the individual microbes.

Community metabolic modelling combines the biochemical networks of multiple microorganisms to predict their interactions and metabolic capabilities in a community setting. This approach aims to simulate the metabolic behaviour of microbial communities in various environments and gain insights into their structure and function. Hence, the microbial GEMs were combined into a compartmentalized community model to understand the altered metabolic behaviour of microbial consortia in healthy and T2D gut environments. The reconstructed community model encompasses 3578 genes, 4163 reactions, and 4399 metabolites in four microbe-specific compartments (Fig. 1a). The individual microbial biomass reactions served as precursors for the community biomass reaction. The biomass production of the community metabolic model was optimized by maximizing the biomass reaction under average human diet (AHD) media (Table S3). The AHD media was designed to mimic the biochemical condition of the human gut microenvironment and can provide a more realistic view of the metabolic flux distribution of the microbial community model. The community model showed a growth rate of 0.33 h^−1^ in the AHD media (Fig. 3). Further, to understand the condition-specific metabolic activity, microbial abundance with respect to healthy and T2D gut environments was implemented to weigh the biomass precursor coefficient in the community model (Table S4). The healthy and T2D microbial community models showed a growth rate of 0.32 h^−1^ and 0.47 h^−1^, respectively.

The community model primarily consumes glucose and amino acids from the AHD media in both healthy and T2D conditions. The T2D gut microbiota exhibits a significant decrease in glucose uptake rate by 39.34% and an increase in amino acid consumption rate by 18.82% compared to the healthy control. The variations in the consumption rate of glucose and amino acids, as predicted by our community model, can alter the concentration of these biomolecules in the human body fluid. The imbalance in glucose and amino acid concentrations within the human body poses a potential risk for Type 2 Diabetes, as highlighted in experimental studies^47^. On the other hand, the community model within the T2D gut environment reveals an altered metabolic exchange pattern between individual microbes when compared with the healthy microbial community. The altered metabolic exchange pattern was accompanied by shifts in the metabolic flux through the biochemical networks of the microbial community. In contrast to the healthy gut microbiota, the microbial community associated with the T2D gut environment exhibited diverse flux rates in reactions involving amino acids, SCFAs, vitamins, alcohols, CO_2_, and bicarbonate exchange between the individual microbes present in the community model. Moreover, the community model in the T2D gut environment also showed reduced production of SCFAs, vitamins, and bicarbonates by 64.32%, 5.54%, and 62.5%, respectively. As the SCFAs and vitamins are essential biomolecules that regulate a vast range of metabolic processes, a reduction in their production flux leads to metabolic dysbiosis in the T2D gut environment. Moreover, the alteration in the bicarbonate concentration can hamper carbohydrate metabolism, which has a considerable effect on the T2D condition. Further analysis of the community model will help comprehend the interconnection between the microbial consortia and assist in predicting the altered biochemical activities of inter-microbial metabolic interactions in the T2D gut environment.

### Flux space alteration within the biochemical pathways of healthy control and T2D gut microbiota community metabolic model

**Figure 4:**
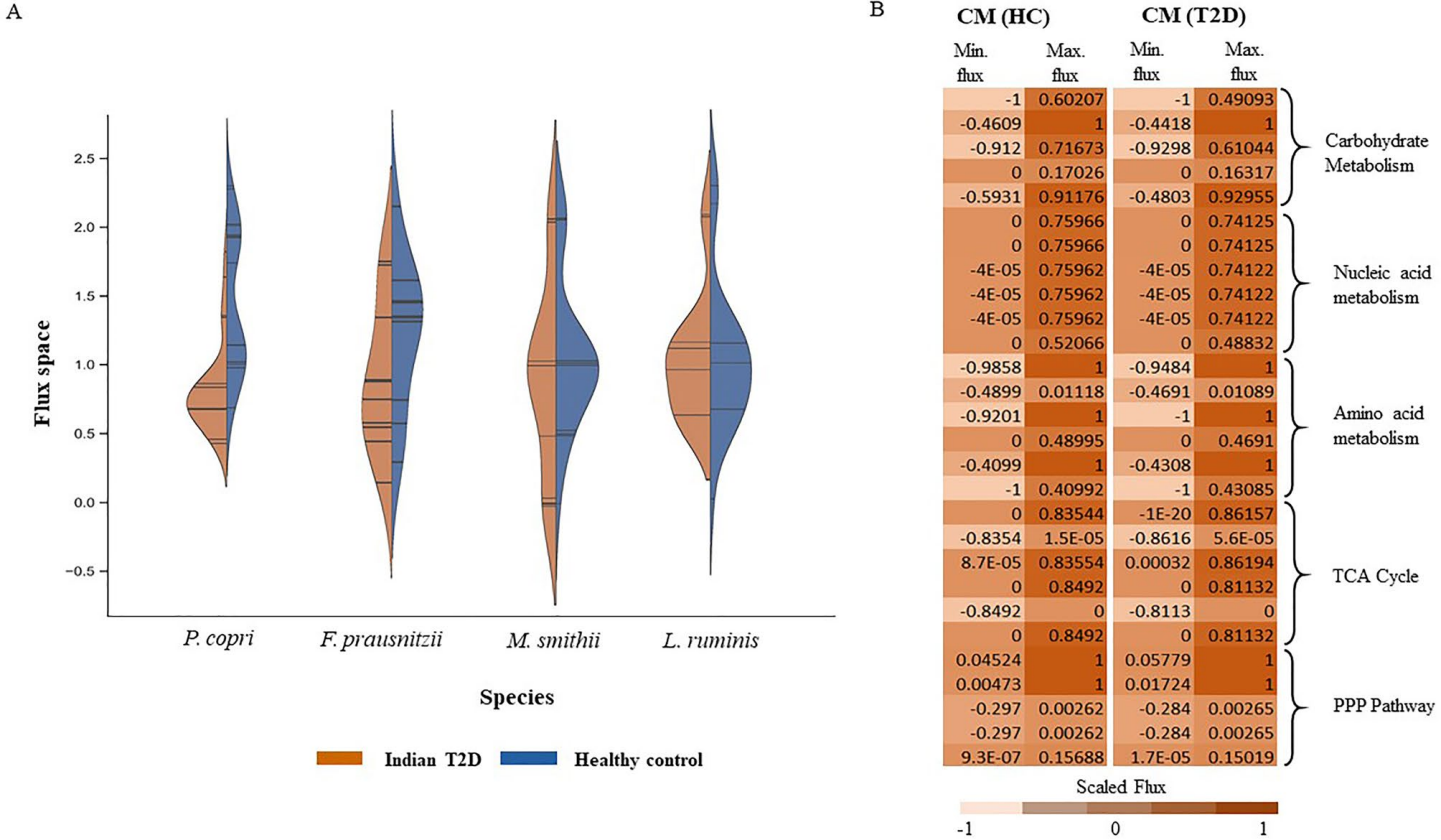
Altered reaction flux range from healthy control gut microbiota community model to T2D community metabolic models. (**a**) Alteration of the reaction flux range between the T2D community model (blue) and the healthy control community model (orange) of the human gut microbiota. The vertical axis represents the flux range, and on the horizontal axis, each point represents the individual microbes. (**b**) The alteration of reaction flux range of several critical metabolic pathways like, carbohydrate metabolism, nucleotide metabolism, amino acid metabolism, TCA cycle and PPP pathway between the healthy control and T2D gut microbiota community model.

**Figure 5:**
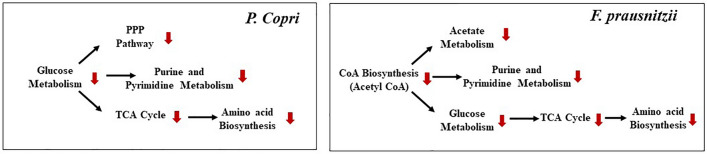
FVA reveals altered microbial biochemical pathways in T2D gut microbiota compared to healthy control. The altered flux space in the biochemical pathways associated with glucose metabolism in *P. copri* and *F. prausnitzii*. The red arrows indicate the decrease in the flux space.

An imbalance in the gut microbial composition can influence the metabolic flux profile of the gut microbiota. Several experimental studies have reported alterations in the gut microbial composition in individuals with Type 2 Diabetes^9^. To understand how the altered microbial composition can influence the metabolic flux profile in T2D gut microbiota, the Flux Variability Analysis (FVA) was performed. The changes in flux space through the biochemical network of a microorganism can lead to the increase or reduction of certain metabolic activities in the microbe. FVA elucidates the fluctuations in the permissible flux range and enables the determination of condition-specific flux space alteration in metabolic models. Analyzing alterations in flux space through the metabolic reactions of each microorganism in the community helps to identify the crucial microbe-specific metabolic pathways that are affected in the T2D condition. The FVA showed an average of ~ 12.50% of reactions have decreased flux space and ~ 5.61% of reactions have increased flux space in the community model in the T2D compared to the healthy gut environment (Fig. 4, Table S5). In the community model, the decreased flux space was associated with different biochemical pathways like pantothenate and CoA biosynthesis, carbohydrate, amino acid, and fatty acid metabolism. Pantothenate (vitamin B5) biosynthesis is an essential checkpoint for carbohydrate metabolism as it plays a vital role in coenzyme A (CoA) production, an essential cofactor in different crucial biochemical reactions. The community model in the T2D condition showed a reduced flux space in pantothenate biosynthesis by 41.44%, 29.36%, and 5.92% in *P. copri*, *F. prausnitzii*, and *M. smithii*, respectively. The distorted pantothenate metabolism of these two community members impaired the CoA biosynthesis of the respective microbes, ultimately affecting glucose metabolism. Therefore, the flux space of biochemical reactions associated with glucose metabolism in the community model has exhibited a reduction of 32.19%, 16.25%, and 4.32% in *P. copri*, *F. prausnitzii*, and *M. smithii*, respectively. Low glucose metabolism by the gut microbiota leads to a high concentration of unutilized glucose retained in the body fluid, which triggers the T2D condition. For instance, the crucial reaction of glucose metabolism that converts phosphoenolpyruvate to pyruvate was reduced by 45.23%, 34.87%, and 21.61% flux space in *P. copri*, *F. prausnitzii*, and *M. smithii* models, respectively. The formation of pyruvate from glucose marks the initiation of various biochemical pathways, including TCA, amino acid, and fatty acid metabolism. Hence, the reduction of glucose metabolism restricts the carbon flow in the subsequent metabolic pathways, which alters the flux distribution in amino acid and fatty acid biosynthetic pathways (Fig. 5). The community model shows a significant reduction of flux space in amino acid biosynthesis, specifically BCAAs, including valine, leucine, and isoleucine. The biochemical reactions associated with BCAA metabolism showed 9.65%, 12.16%, and 4.32% reduced flux space in *P. copri*, *F. prausnitzii*, and *M. smithii*, respectively. Specifically, the metabolic reaction converting glutamate into leucine showed a reduced flux range of 6.19% in *M. smithii* (Table S5). This shift in the flux space within the BCAA metabolism can lead to an altered biosynthesis of the respective amino acids, which can give rise to various health complications and elevate the risk of T2D, as demonstrated in multiple epidemiological studies^48–52^. Apart from the amino acid metabolism, the altered flux flow in the carbohydrate metabolism also affected the fatty acid metabolism biosynthesis in the T2D community environment. An 8.23%, 56.19%, and 84.23% reduction in the SCFA metabolism (specifically acetate and butyrate) was observed in *P. copri*, *F. prausnitzii*, and *M. smithii*, respectively. Acetate and butyrate are crucial short-chain fatty acids (SCFAs) that help enhance glucose and lipid metabolism^5^. Hence, the reduced concentrations of these fatty acids in the body fluids can impact glucose metabolism and lead to biochemical complications in T2D patients. Overall, the FVA helps to identify crucial biochemical pathways in the microbial community where the flux range was affected due to metabolic dysbiosis in the T2D condition. Moreover, it also provided systems-level knowledge on how the alteration of flux distribution in carbohydrate metabolism can affect the interconnected biochemical pathways and alter community metabolism. Therefore, the interconnectivity of the microbial flux distribution pattern and how it affects overall metabolic outcomes in T2D conditions were addressed in the following section.

### Assessing the altered metabolic activities of the T2D gut microbial community

**Figure 6:**
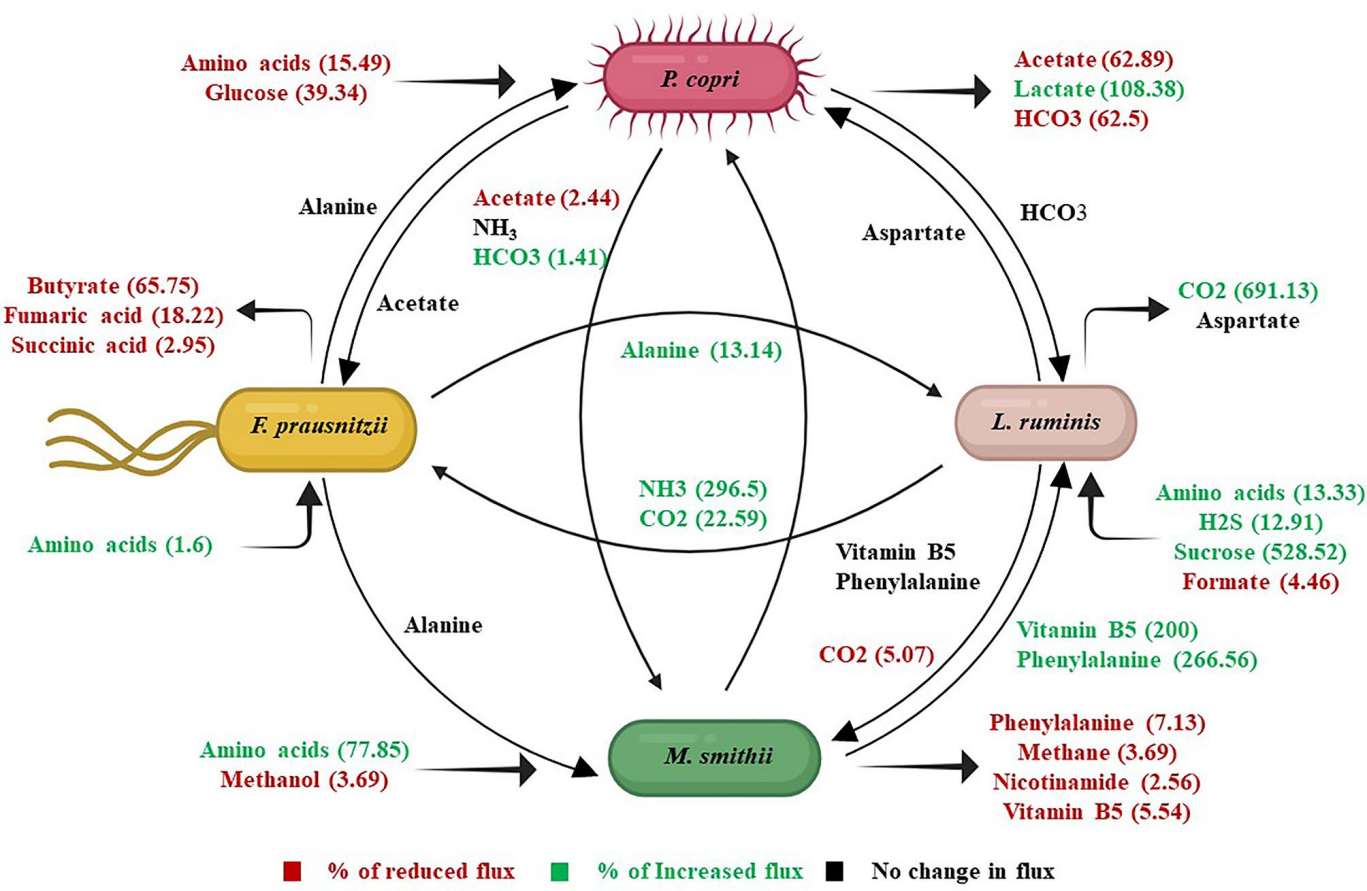
Community-level metabolic interaction between the crucial gut microbiota. (**a**) This network represents the altered metabolic interaction in the microbial community between healthy and T2D gut environments. The values represent the percentage of flux change between two conditions. Red-colored metabolites show decreased flux, green shows increased flux, and black shows no change in flux.

The biochemical pathways of gut microbes, specifically glucose, SCFA, and AA metabolism, were altered in diabetic patients compared to healthy individuals, as determined by flux space analysis. These varied metabolic activities of the gut microbial community can influence the flux distribution pattern of human biochemical pathways, which can play crucial roles in T2D. Therefore, the community-level flux analysis was performed to better understand the altered metabolic activities of the microbial community in the T2D gut environment compared to the healthy control (Table S6). Moreover, the assessment of the pathway-level map helps to explore the metabolic interconnection between human gut microbiota and T2D. The analysis of community-level flux distribution indicates that the microbial community in the human gut predominantly utilizes amino acids, vitamins, short-chain fatty acids (SCFAs), and glucose as primary metabolic substrates. However, varied rates of substrate consumption were noticed in the microbial community in accordance with the specific gut environment (Table S7). This altered substrate consumption influenced both the metabolism of the microbial community and the metabolic interactions among the individual microbes. We also have identified the alteration in the inter-microbial metabolic exchange pattern for different essential metabolites in the community environment. For instance, *P. copri* utilizes glucose as its primary carbon source and generates various short-chain fatty acids (SCFAs) and bicarbonate. A glucose consumption rate of 9.265 mmol/gDW/h was observed for *P. copri* in the community model in the healthy gut environment. In contrast, a reduced glucose intake of 5.62 mmol/gDW/h by *P. copri* was observed in the community model in the T2D gut environment (Fig. 6). As *P. copri* is the only glucose-consuming microbe in the microbial community, the reduction in its glucose consumption rate can increase the glucose concentration in the human body fluid. Consequently, the reduced glucose uptake rate in *P. copri* disrupts the flux profiles of its inherent metabolic pathways, resulting in a decline in biochemical activities related to SCFAs and bicarbonate metabolism. The production of major SCFA, namely acetate, was reduced by 62.89% in the T2D gut microbial environment compared to the healthy condition. The reduced production of SCFA impacts the inter-microbial exchange and cross-feeding of acetate—between *P. copri* and *M. smithii* in the community environment. In the T2D condition, the exchange of acetate between this microbial pair was decreased by 2.44%, respectively. The reduced production and exchange of acetate and formate eventually leads to a lower accumulation of these SCFAs in the gut environment. As acetate is one of the major SCFAs that maintain intestinal mucosa integrity^4,13^ and improve glucose and lipid metabolism^5^, a reduction in their concentration within the T2D gut environment may impair glucose metabolism, thereby influencing blood sugar levels. Furthermore, the acetate produced by *P. copri* also acts as a major substrate for *F. prausnitzii* in the microbial community*.* By getting assistance from the gut microbial community, *F. prausnitzii* produces butyrate. Interestingly, it was observed that *F. prausnitzii* shows a reduced production of butyrate by 65.75% in the T2D gut environment compared to the healthy control. Different experimental studies have reported that butyrate has a similar effect as metformin in reducing insulin resistance and improving glucose metabolism^53^. Therefore, the reduced concentration of butyrate can play a crucial role in impaired insulin sensitivity and reduced glucose metabolism. In addition to SCFAs, *P. copri* also produces bicarbonate, which *L. ruminis* and *M. smithii* subsequently consume as a metabolic substrate. *P. copri* and *M. smithii* exhibited an elevation of 1.41% in bicarbonate exchange in the gut environment of T2D as compared to healthy control. The increased bicarbonate consumption by *M. smithii* in the community environment led to a significant decrease (62.5%) in bicarbonate accumulation in the T2D gut microbiota compared to the healthy environment. The decreased bicarbonate concentration disrupts insulin secretion, which can lead to altered glucose regulation in T2D patients, as mentioned in earlier studies^54^. Moreover, the lower bicarbonate flux also restricts the carbon flow to the carbohydrate metabolism pathway and ultimately hampers the metabolic homeostasis in the T2D condition.

Several other critical metabolic byproduct exchanges, including phenylalanine, vitamin B5, and CO_2,_ have also been identified in the T2D community environments. *M. smithii* acts as the primary producer of these metabolic compounds, which *L. ruminis* consumes in the community environment. *F. prausnitzii*, along with *M. smithii*, provides positive metabolic assistance to *L. ruminis*, resulting in its higher abundance in the T2D gut environment. This increase in the abundance of *L. ruminis* in the microbial community showed an enhanced sucrose consumption of 528.52%. Sucrose is a prominent substitute for glucose. An increase in sucrose consumption has resulted in a 39.34% reduction in glucose intake. Hence, this high consumption of sucrose and low glucose intake rate result in an elevation of glucose concentration in the body fluid, potentially increasing complications in T2D.

Similarly, the metabolic exchange profiles of vitamin B5 have also been altered in the T2D gut environment. Overall, a 6% reduction in the production of vitamin B5 was found in the T2D gut environment compared to the healthy control. These lower vitamin B5 production profiles impacted the exchange flux between *M. smithii*, *L. ruminis,* and *P. copri,* leading to an overall 5.54% decrease in vitamin B5 concentration in the body fluids. As vitamin B5 acts as an essential precursor for CoA biosynthesis, the reduced concentration in the human body can hinder protein, carbohydrate, and lipid metabolism in T2D, as pointed out in earlier studies^55,56^.

Consequently, the community model in the T2D gut environment also showed altered concentrations of various BCAAs, where the consumption of valine, leucine, and isoleucine decreased by 9.23, 15.16%, and 8.29%, respectively (Fig. 6). The reduction in amino acid intake by the microbial community implies an elevation in BCAA levels within the human body fluid, which has also been reported in earlier experimental studies of type 2 diabetes patients^52,57^. Hence, the community-level metabolic flux analysis reveals that maintaining the concentration of several critical metabolites, like SCFAs, BCAAs, vitamins, and bicarbonate, in the body fluids is critical in managing T2D complications. The alteration in the concentration of these essential metabolites in the body fluids can be conceded as biomarkers for Indian T2D. Moreover, the metabolic exchanges between crucial gut microbial species help to balance the metabolite concentration and biochemical homeostasis in the body. Hence, the disruption of the microbial community composition and abundance profiles has a significant impact on worsening the T2D condition. The systems-level knowledge of the microbial community-level flux distribution derived from this study can help manipulate and optimize the microbial biochemical activities for future therapeutic implications.

## Conclusion

The genome-scale metabolic models of crucial Indian T2D gut microbes, i.e., *P. copri*, *L. ruminis, M. smithii,* and *F. prausnitzii,* were constructed to elucidate the intricacies of microbial metabolism in diabetic patients. The individual microbial GEMs were combined to create the genome-scale community model. In order to assess and compare the metabolic landscape of the healthy and T2D gut environments, the biomass production of the community model was optimized considering the microbial composition and biochemical profiles of the respective environments. The community model in the T2D gut environment showed reduced glucose consumption and metabolism in the gut microbiota. The reduction in glucose intake can lead to the retention of excess glucose in the body fluid, which is a major cause of T2D. The elevation in glucose concentration can profoundly affect human organelles, potentially triggering the onset of numerous diseases^58^. In addition to glucose, the production of SCFAs like acetate and butyrate was reduced in the T2D gut microbiota. Acetate is a major short-chain fatty acid that maintains intestinal mucosal integrity^4^ and improves glucose and lipid metabolism^5,13^. Hence, the decrease in acetate concentration can also hamper glucose metabolism. Furthermore, community modelling of the human gut microbiota showed a decreased butyrate concentration in T2D patient**’**s body fluid. Butyrate serves as a histone deacetylase inhibitor and binds to G-protein-coupled receptors, influencing β-cell growth, apoptosis, and insulin secretion in the human pancreas^59^. Consequently, the decreased levels of butyrate have significant health implications for T2D patients and can be considered a therapeutic target. Similarly, the consumption of several amino acids like BCAAs and AAAs was reduced in the gut environment, which can lead to a retention of excess amino acids in the human body. Thus, the increased concentration of BCAAs and AAAs may act as a biomarker for T2D. Moreover, this study demonstrated a diminished production of bicarbonate within the gut microbiota environment of individuals with T2D, resulting in a decreased bicarbonate presence in human bodily fluids. The reduction in bicarbonate concentration can impede the flow of carbon to the carbohydrate metabolism pathway, thereby disrupting metabolic homeostasis in individuals with T2D. Further, the community model showed a reduced production of vitamin B5 (pantothenic acid), which leads to a reduction of this compound in the body fluid. Pantothenic acid is the precursor molecule for CoA biosynthesis, so a lower concentration of pantothenic acid may impact human cellular metabolism^55^. The study revealed crucial metabolic interlinks between the human gut microbiota and Indian T2D. Moreover, the study demonstrated that *P. copri and F. prausnitzii* play a significant role in gut microbiota metabolism and can influence human metabolic pathways by altering the concentration of different metabolites in the body fluids, which include SCFAs, AAs, glucose, and bicarbonate. Therefore, *P. copri and F. prausnitzii* could serve as probiotics to help maintain the homeostasis of the gut microbiota, potentially leading to improved health in T2D patients. Overall, the proposed community modelling approach and in silico flux analysis have elucidated the metabolic dynamics of the human gut microbiota in diseased and healthy states. This method has provided a comprehensive understanding of how alterations in the composition of the T2D gut microbiota and their corresponding metabolic activities can affect the underlying physicochemical conditions in the diseased state. Therefore, the flux-level insights gained from the community metabolic model may lead to the strategic implementation of personalized interventions, such as tailored dietary regimens and targeted microbiome therapies with probiotic and antibiotic treatments. The controlled modulation in the biochemical profile of the gut microbiota might pave new avenues for improvising the strategy of T2D management and treatment.

## Methodology

### Genome-scale model reconstruction and refinement

The whole-genome sequences of *Prevotella copri*, *Lactobacillus ruminis, Methanobrevibacter smithii,* and *Faecalibacterium prausnitzii* were acquired from the NCBI. The microbe-specific annotated genome was used to construct the draft GEMs using the modelSEED server^60^. The draft models had reaction gaps, unbound reactions, and charge and mass imbalance, which needed to be corrected for better prediction (Tables S1, S2). The manual curation of all the GEMs was performed by using different reference databases like the KEGG^61^, ModelSeed^60^, PubChem, BiGG^44^, and the reported research knowledge. The individual model was enhanced with various microbe-specific metabolic reactions using the available genetic information. The draft models were curated using COBRApy tool^62^ with Gurobi Optimizer version 9.5.2 in SBML format^63^.

### Biomass sensitivity analysis

Sensitivity analysis checks how a small alteration in the coefficient of biomass precursors can affect the specific growth rate of an individual microbe. For sensitivity analysis, the coefficient of biomass precursor molecules was individually changed by ± 20% one at a time. Subsequently, FBA^64^ was employed to check the growth rate of the individual microbe as follows^65^:

$$\begin{gathered} Maximize\left( {v_{j} } \right){\text{d}}^{*T} v_{biomass} \hfill \\ Subjected\;to = { }\mathop \sum \limits_{{{\text{j}} \in {\text{J}}}} {\text{S}}_{{{\text{ij}}}} {\text{V}}_{{\text{j}}} = 0\quad \forall i \in I \hfill \\ lower\;bound \le v_{j} \le upper\;bound_{j} \forall i \in I \hfill \\ \end{gathered}$$

Here, d* is the changed biomass precursor coefficient, d* = (d1, d2, d3 …. dn ± 0.01dn). Here, the biomass precursor ‘dn’ coefficient was adjusted by ± 20% by keeping others unchanged.

### Community metabolic model reconstruction

The GEMs of *P. copri, L. ruminis*, *M. smithii,* and *F. Prausniizii* were combined into the community model using a compartmentalized approach. Each model’s genes, reactions, and metabolites were assigned a unique compartment with a specific annotation during individual model integration. For example, in the case of *P. copri* all the reactions and metabolites were assigned an extension of ‘pc’, which is true for all the models. So, all the GEMs can represent their unique compartments in the multi-species community model and have common extracellular space. This enables the metabolic exchange among the individual species, as all the microbes share the same space. All the metabolic compounds consumed or produced by the microbes in the extracellular space were annotated with an extension ‘e0’. To define the community biomass, biomass objective functions from all individual species models were integrated by summing their stoichiometric coefficients using the following optimization formula^66^.

$$Z = \max \left( {v_{biomass}^{community} } \right),$$

$$Community\, Biomass = \mathop \sum \limits_{k = 1}^{N} a_{k} *v_{biomass}^{k}$$

Here, “Z” is objective function, $"{v}_{biomass}^{community}"$ represents the community biomass reaction and “a_k_” is the abundance of *P. copri*, *L. ruminis*, *M. Smithii*, and *F. prausnitzii* in the T2D and HC gut environments. With this approach, a large number of individual metabolic models can be integrated into a community metabolic model based on their meta-genomics abundance. The biomass coefficient of GEMs as the precursor of the community biomass reaction was updated based on the metagenomics data (Table S4), which can reflect the community environment more accurately. The community model can show the inter-microbial metabolic activities and the community metabolic flux profile in a quasi-steady state condition.

### Flux balance analysis

FBA is a mathematical modelling technique used in systems biology to analyze and predict the flow of metabolites through a biochemical reaction. It is based on the principle of mass balance, where the rates of metabolite production and consumption are balanced to achieve a steady-state condition. Flux balance analysis was performed at each stage of model refinement, analysis of fluxes, deciding upper and lower bounds, and gap filling. The algorithm for performing flux balance analysis follows:

$${\text{Maximize}}\;{\text{v}}_{{{\text{biomass}}}} \;{\text{subject to}}$$

1 $$\mathop \sum \limits_{{{\text{j}} \in {\text{J}}}} {\text{S}}_{{{\text{ij}}}} {\text{V}}_{{\text{j}}} = 0\forall_{i} \in I$$

2 $${\text{LB}}_{{\text{j}}} \le {\text{v}}_{{\text{j}}} \le {\text{UB}}_{{\text{j}}} \forall_{{\text{j}}} \in I$$

where i = Metabolite, j = Reaction, S = stoichiometric coefficients, V = fluxes, LB = Lower bound of reaction, UB = Upper bound of reaction.

### Flux variability analysis

The flux space of a biochemical reaction from an individual entity of a community model can significantly change with the alteration in the composition of the community. FVA ^67^ generates the flux space for all reaction flux *v*_*i*_ in the community model by solving linear programming problems in a given optimal state (*Sopt*):

$$\begin{gathered} Maximize\;or\;minimize\;v_{i} \hfill \\ Subjected\;to{:}S = Sopt \hfill \\ S.v = 0 \hfill \\ and\;v_{i} ,\min \le v_{i} \le v,\max ,\;for\;i = 1,2,3, \ldots n \hfill \\ \end{gathered}$$

The flux space can be compared to the community model associated with the different environments. FVA helps to identify the expansion of metabolic flux in different biochemical pathways in separate compartments of the community.

### Supplementary Information

Supplementary Tables.

## Data Availability

The authors declare that all other relevant data are available within the article and its supplementary data files. The associated models and codes are available on Git Hub (https://github.com/itsamit/Type-2-diabetes).
